# Experimental atopic dermatitis depends on IL-33R signaling via MyD88 in dendritic cells

**DOI:** 10.1038/cddis.2017.90

**Published:** 2017-04-06

**Authors:** Changwei Li, Isabelle Maillet, Claire Mackowiak, Camille Viala, Franco Di Padova, Mei Li, Dieudonnée Togbe, Valérie Quesniaux, Yuping Lai, Bernhard Ryffel

**Affiliations:** 1 Experimental and Molecular Immunology and Neurogenetics (INEM), UMR 7355 CNRS and University of Orleans, F-45071 Orleans-Cedex 2, France; 2 Shanghai Institute of Traumatology and Orthopaedics, Shanghai Key Laboratory for Prevention and Treatment of Bone and Joint Diseases with Integrated Chinese-Western Medicine, Ruijin Hospital, Jiao Tong University School of Medicine, Shanghai 200025, China; 3 Novartis Institutes for Biomedical Research WSJ 386.11.48.38 4002, Basel, Switzerland; 4 Institut de Génétique et de Biologie Moléculaire et Cellulaire, Centre National de la Recherche Scientifique UMR7104/Institut National de la Santé et de la Recherche Médicale U964/Université de Strasbourg, Illkirch, France; 5 University of Strasbourg Institute for Advanced Study, Strasbourg, France; 6 Freiburg Institute for Advanced Studies, Freiburg, Germany; 7 Artimmune SAS, rue Bouffon, 45071 Orleans-Cedex 2, France; 8 IDM, Institute of Molecular Medicine, University of Cape Town, Cape Town, South Africa; 9 Shanghai Key Laboratory of Regulatory Biology, School of Life Sciences, East China Normal University, Shanghai 200241, China

## Abstract

Atopic dermatitis (AD) is a chronic Th2 type inflammatory skin disorder. Here we report that MyD88 signaling is crucial in the pathogenesis of experimental AD induced by vitamin D3 analog MC903. The clinical signs and inflammation caused by MC903 are drastically reduced in MyD88^−/−^ mice with diminished eosinophil, neutrophil infiltration and Th2 cytokine expression. The biological effect of interleukin-1 (IL-1) family members relies on MyD88 signaling. We observed a strong upregulation of IL-1 family cytokine expression, including IL-1*α*, IL-1*β*, IL-33, IL-18, IL-36*α*, IL-36*β*, IL-36*γ* and IL-36Ra. Therefore, we asked which cytokine of the IL-1 family would be essential for MC903-induced AD syndrome. We find a significant reduction of AD in IL-33^−/−^ and IL-33R/ST2^−/−^ mice, only a minor reduction in double IL-1*αβ*^−/−^ mice and no difference in IL-36R^−/−^ and IL-36Ra^−/−^ mice. IL-33 is expressed in keratinocytes, and MyD88 signaling in dendritic cells (DCs) is crucial for AD development as inflammation was drastically reduced in DC-specific MyD88^−/−^ mice (CD11c-cre × MyD88-floxed). Taken together, the data demonstrate a critical role of MyD88 in DCs and of IL-33 signaling via ST2 in MC903-induced AD. These data suggest that IL-33/IL-33R may be a therapeutic target of AD.

Atopic dermatitis (AD) is a chronic Th2 type inflammatory skin disease associated with cutaneous hyperreactivity to environmental triggers.^1^ It affects at least 15% of children and 3% of adults.^2^ Patients suffer from relapsing eczematous and occasionally generalized (erythroderma) lesions associated with severe pruritus.^3^ Chronic AD results in the infiltration of inflammatory dendritic cells (DCs), macrophages and eosinophils in the lesions.^4, 5^ This skin disease is frequently associated with other allergic disorders such as asthma, rhinitis or conjunctivitis.^6^ Moreover, AD causing pruritus affects the quality of life of patients and increases the susceptibility to microbial colonization such as *Staphylococcus aureus* infections.^6, 7^ This is why AD remains a serious health concern in many countries today. The diagnosis of AD is based on the clinical signs of itching, facial and extensor eczema in infants and children, flexural eczema in adults, and chronicity of dermatitis.^4^ However, the underlying pathophysiological and genetic mechanisms leading to the manifestation of AD need further investigations.

The clinical phenotypes that characterize AD are skin barrier dysfunction and immune dysregulation. Filaggrin gene mutation leads to skin barrier dysfunction and transepidermal water loss, resulting in an AD syndrome.^8, 9^ The most cited explanations for this allergic disease are an increase in serum immunoglobulin E (IgE) as well as T helper-2 (Th2) immune responses with increased interleukin-4 (IL-4), IL-5, IL-10 and IL-13.^6, 7, 10^ These phenomena lead to increased allergens exposure, which are picked up by Langerhans cells to lymph nodes and stimulate naive CD4^+^ T cells (Th0) to differentiate into Th2 cells. The associated cytokines, which are produced such as IL-4 and IL-13, are known to stimulate the production of IgE, whereas IL-5 is one of the most important cytokines for generation of eosinophils. A well-established function of IgE is its mediation in mast cells activation,^11^ which can induce the expression of proinflammatory cytokines by keratinocytes as well as migration of DCs. It has been reviewed that impaired barrier functions could stimulate signaling cascades, engaging an epidermal homeostatic response, as well as a type 2 inflammatory reaction.^12^

The IL-1 family is a group of cytokines that has a central mediator of innate immunity and inflammation. It is constituted by three subfamilies: IL-1 subfamily (IL-1*α*, IL-1*β* and IL-33), IL-18 subfamily (IL-18 and IL-37) and IL-36 subfamily (IL-36*α*/*β*/*γ*, IL-36Ra and IL-38).^13^ All IL-1 family members binding to their specific receptors recruiting the shared adaptor protein MyD88 and IL-1R-associated kinase IRAK, have a major pathogenic role in autoinflammatory, autoimmune, infectious and degenerative diseases.^13, 14, 15, 16^ For the function of IL-1 family cytokines in the pathogenesis of AD, recent data highlighted increased IL-33 in skin of patients with AD and in ovalbumin-induced experimental AD.^17^ In addition, Konishi *et al.*^18^ revealed the skin specifically overexpressed IL-18 led to spontaneous development of AD-like inflammatory skin lesion. Furthermore, it has been reported that IL-1 enhances AD development.^18^ The role of other members of the IL-1 family in AD induction, such as IL-36*α*/*β*/*γ* or its antagonist IL-36Ra, is poorly understood. There is no report correlating AD severity of inflammation between IL-33 and other IL-1 family member deficient mice.

The vitamin D3 analog MC903, used to treat patients with skin cancer, has been reported to induce an AD-like syndrome, including Th2 type inflammation with eosinophilia and hyper-IgE immunoglobulinemia.^19, 20, 21^ In order to understand the function of IL-1 family cytokines in the pathogenesis of AD, we used MC903 to induce AD in mice. First, we investigated the expression of the different IL-1 members in MC903-induced AD and then compared the severity of AD inflammation in BL6 and in gene-deficient mice including IL-1*αβ*^−/−^, IL-1R1^−/−^, IL-36 R^−/−^, IL-36Ra^−/−^, IL-33^−/−^, IL-33 receptor ST2^−/−^, MyD88^−/−^, DC-specific MyD88^−/−^ (CD11c-cre × MyD88-floxed) and T-cell-specific MyD88^−/−^ (CD4-cre × MyD88-floxed) mice. We uncovered a critical role of MyD88 in DCs and identified IL-33 signaling via ST2/MyD88 to be critically involved in AD development.

## Results

### The vitamin D3 analog MC903 induces experimental AD

**Figure 1:**
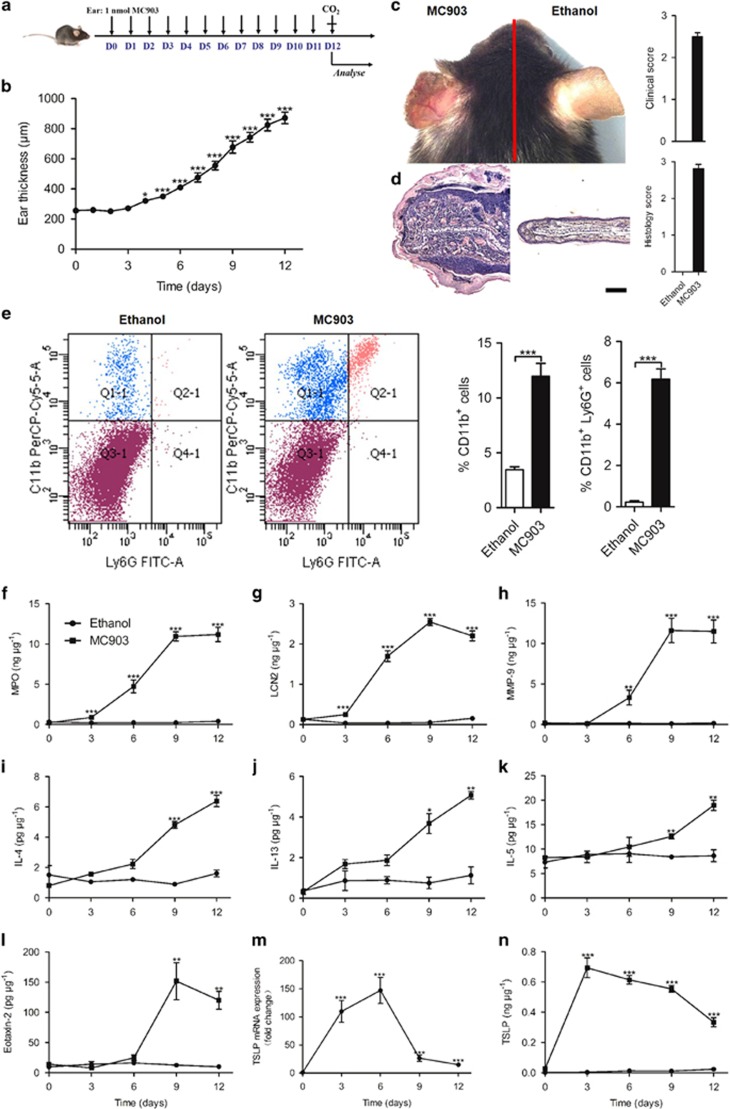
Vitamin D3 analog MC903 induces AD. (**a**) Schematic of MC903 application. (**b**) Increase in ear thickness after daily MC903 treatment. (**c** and **d**) Photographs (**c**) and H&E staining (**d**) of MC903- and ethanol-treated ears at day 12. Scale bar represents 50 *μ*m. (**e**) The infiltrating CD11b^+^ and CD11b^+^ Ly6G^+^ cells in skin of AD lesions by FACS. (**f**-**n**) The expression of MPO (f), LCN-2 (**g**), MMP-9 (**h**), IL-4 (i), IL-13 (**j**), IL-5 (**k**), eotaxin-2 (**l**) and TSLP (**m** and **n**) in the ear treated with ethanol or MC903. *P<0.05,**P<0.01,***P<0.001. *P*-values were analyzed by one-way ANOVA in **b** and **m**, two-tailed *t*-tests in **e** or two-way ANOVA in **f**-**l** and **n**. All data are representative of two to three independent experiments with *n*=5 per group and are means±S.E.M.

In order to better understand the underlying pathophysiologic and genetic mechanisms of AD, we used the vitamin D3 analog MC903 to induce AD in mice. The daily topical administration of MC903 on the ear caused swelling, reddening and scaling of the skin (Figures 1a and c). Microscopic investigation revealed a strong dermal inflammatory cell infiltration with epidermal hyperplasia and hyperkeratosis (Figure 1d). Abundant myeloid cell and lymphocyte infiltration 9 days after topical MC903 application as compared with ethanol-treated control mice was identified by flow cytometry analysis (Figure 1e). MC903-induced neutrophil recruitment and activation as evidenced by increased myeloperoxidase (MPO), lipocalin-2 (LCN-2) and metalloproteinase-9 (MMP-9), which are detectable at day 3 and reaching a maximum at day 9 (Figures 1f and h). These results are consistent with reports on chronic AD.^4, 5^ As Th2 cytokines are considered to be involved in the pathogenesis of AD, we found augmented IL-4, IL-5 and IL-13 in the inflamed skin by ELISA within 3 days, which increased up to day 12 (Figures 1i and k). Furthermore, patients with AD displayed elevated blood eosinophil counts and plasma levels of IL-5, which has an important role in eosinophils development and survival.^22^ Consistent with this report, we found increased expression of eotaxin-2 within 6 days, peaking at 9 days, indicating eosinophil activation (Figure 1l). Furthermore, the expression of thymic stromal lymphopoietin (TSLP), which is involved in MC903-induced AD development,^20, 23^ was also induced in a time-dependent manner (Figures 1m and n). Therefore, MC903 induces a robust AD-like allergic inflammatory response.

### IL-33 is upregulated by epithelial cells in skin upon MC903-induced AD

**Figure 2:**
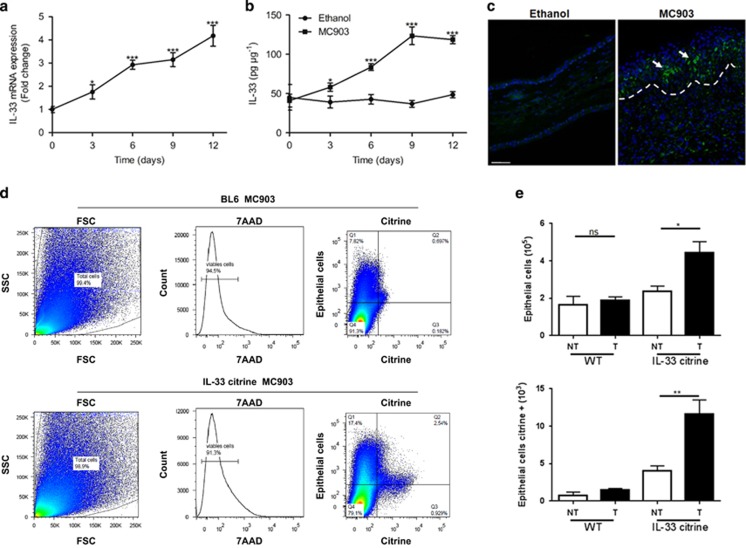
IL-33 is mainly expressed by epithelial cells and CD11b^+^ cells. (**a** and **b**) Quantification of IL-33 mRNA (**a**) and protein (**b**) expression in the BL6 mouse ears for different times. (**c**) Immunofluorescence analysis of IL-33 in mouse ear skin after 12 days of MC903 treatment. This image is representative of five mice with abscess. Scale bar represents 50 *μ*m. (**d** and **e**) Expression of total and citrine Epcam^+^ cells assessed by flow cytometry of MC903 administrated BL6 and IL-33 citrine reporter mice at day 12. *P<0.05, **P<0.01,***P<0.001. *P-*values were analyzed by one-way ANOVA in **a** and **e**, two-way ANOVA in **b**. All data are representative of two independent experiments with *n*=3–5 per group and are means±S.E.M.

As IL-1 family cytokines function as a central mediator of innate immunity and inflammation, we investigated the expression of IL-1 family cytokines in skin, and found a significant upregulation of IL-33 expression at both gene and protein levels upon MC903 administration (Figures 2a and b). Immunofluorescence analysis using antibody to IL-33 revealed that IL-33 was strongly expressed in the nucleus of epithelial cells in ethanol-treated controls, which was drastically upregulated after 12 days of MC903 treatment. However, IL-33 was undetectable in MC903-treated IL-33-deficient mice (data not shown). To determine which cell types express IL-33 in AD, we used IL-33 citrine reporter mice using flow cytometry. IL-33^+^ cells in the skin were essentially epithelial cells, MC903 increased IL-33^+^ citrine expression in keratinocytes (Figure 2d), whereas only a small number of myeloid cells expressed IL-33 (data not shown). Furthermore, IL-33 was mainly expressed in Epcam^+^ epithelial cells, whereas neutrophils, macrophages and DCs represented minor cell populations (Figure 2e). Finally, besides IL-33, MC903 also induced IL-1*α*, IL-1*β*, IL-36*α*, IL-36*β*, IL-36*γ*, IL-36Ra and IL-18 gene expression in a time-dependent manner (Supplementary Figure S1). Therefore, IL-33 expression was induced essentially in epithelial cells of MC903-induced AD.

### The development of AD by MC903 is dependent on IL-33 signaling

Given that MC903 induced high expression of IL-1 family cytokines, we compared the AD severity of inflammation in BL6, IL-1R1^−/−^, IL-1*αβ* ^−/−^, IL-36 R^−/−^, IL-36Ra^−/−^, IL-33^−/−^ and IL-33 receptor ST2^−/−^ mice. No significant difference was found between BL6, IL-36 R^−/−^ and IL-36Ra^−/−^ mice after 12 days of topical MC903 administration either on inflammatory cell infiltration or the cytokine expression (Supplementary Figures S2 and S3). Only a minor decrease of ear swelling and IL-13 expression in IL-1*αβ*^−/−^ mice and IL-4 expression in IL-1R1^−/−^ mice was detected as compared with BL6 mice (Supplementary Figure S4a), but no difference was found on inflammatory cell infiltration and clinical score between BL6, IL-1*αβ*^−/−^ and IL-1R1^−/−^ (Supplementary Figures S4b-S4c and S5b-S5c) with a nonsignificant decrease of MPO, MMP-9 and eotaxin-2 (Supplementary Figures S4e-S4h and S5e-S5h) in IL-1*αβ*^−/−^ and IL-1R1^−/−^ as compared with BL6 mice.

**Figure 3:**
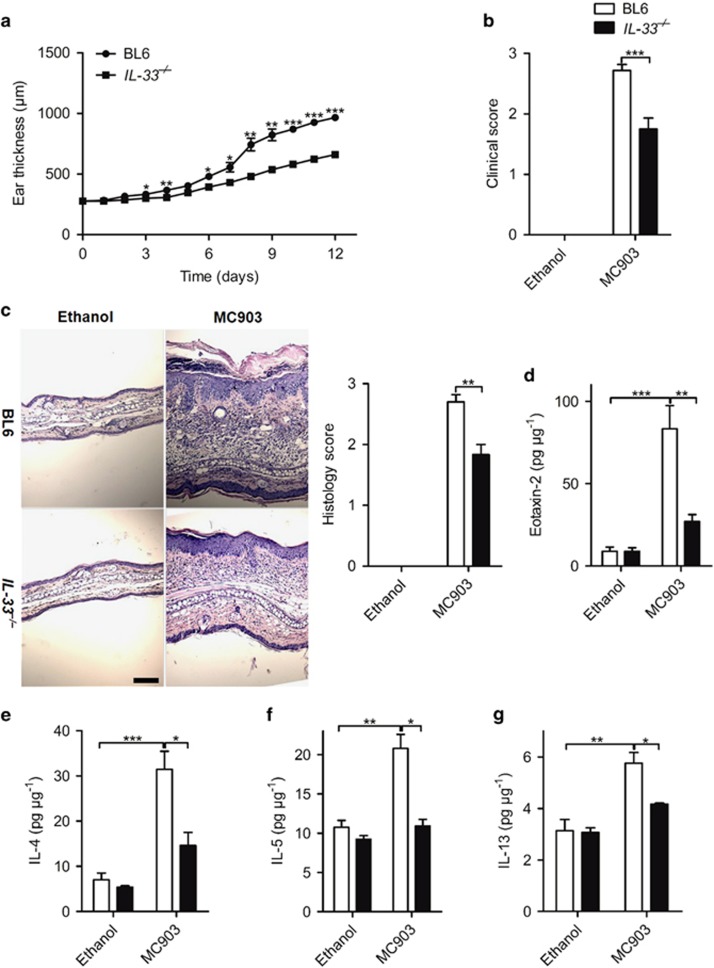
IL-33 is essential for MC903-induced AD. (**a**) Ear thickness of BL6 and IL-33^−/−^ mice after daily MC903 treatment. (**b** and **c**) Clinical score of the ear redness and scaling (**b**) and H&E staining (**c**) of the ear and of BL6 and IL-33^−/−^ mice treated with ethanol or MC903 at day 12. Scale bar represents 50 *μ*m. (**d**-**g**) The protein expression of eotaxin-2 (**d**), IL-4(**e**), IL-5 (**f**) and IL-13 (**g**) in the ear of BL6 and IL-33^−/−^ mice treated with ethanol or MC903 at day 12. *P<0.05,**P<0.01,***P<0.001. *P*-values were analyzed by two-way ANOVA in **a** and one-way ANOVA in **b**-**g**. All data are representative of two to three independent experiments with *n*=4–8 per group and are means±S.E.M.

By contrast, MC903-induced AD syndrome was markedly decreased in IL-33^−/−^ mice with diminished ear swelling (Figure 3a), clinical score (Figure 3b), inflammatory cell infiltration (Figure 3c), eosinophil activation-related protein eotaxin-2 and Th2 cytokine expression (Figures 3d and g). In conclusion, the data demonstrate that MC903 upregulated IL-1 family cytokines, but only IL-33 appears to be essential for MC903-induced AD syndrome.

### IL-33 mediates MC903-induced AD via ST2-MyD88 signaling

Having identified IL-33 is essential for MC903-induced AD syndrome, we explored the molecular mechanisms involved in the IL-33- dependent AD development.

**Figure 4:**
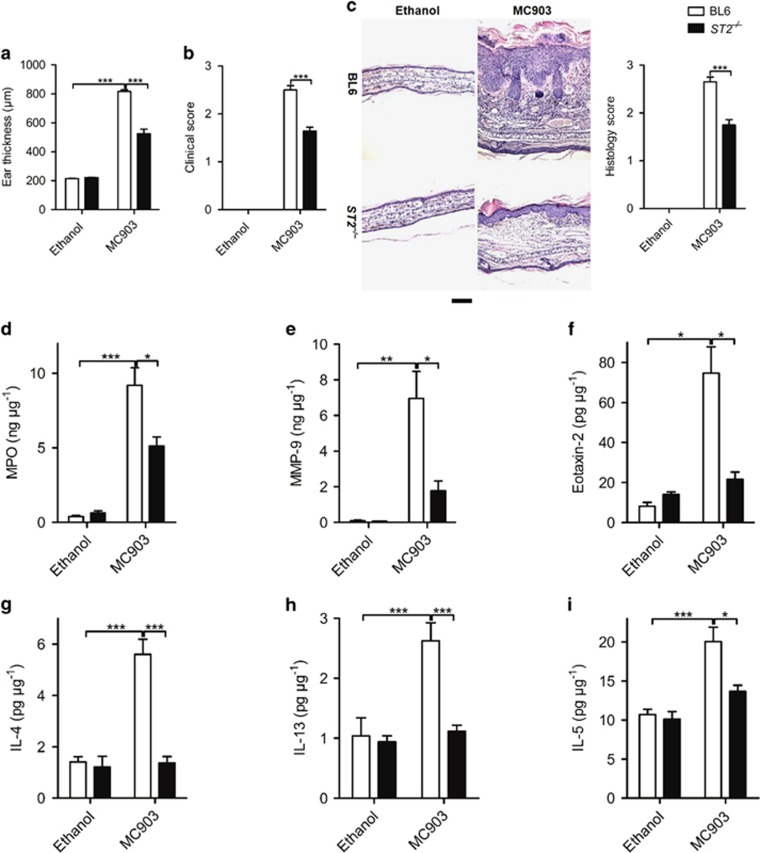
IL-33 mediates MC903-induced AD via ST2 pathway. (a-c) The increase in ear thickness (**a**), clinical score of the ear redness and scaling (**b**) and H&E staining of the ear (**c**) of BL6 and ST2^−/−^ mice after 12 days of MC903 treatment. Scale bar represents 50 *μ*m. (**d**-**i**) The protein expression of MPO (**d**), MMP-9 (**e**), eotaxin-2 (**f**), IL-4 (**g**), IL-13 (**h**) and IL-5 (**i**) in the ear of BL6 and ST2^−/−^ mice treated with ethanol or MC903 at day 12. *P<0.05, **P<0.01,***P<0.001. *P*-values were analyzed by one-way ANOVA. All data are representative of two to three independent experiments with *n*=4–8 per group and are means±S.E.M.

First, we found MC903-induced AD was markedly decreased in ST2^−/−^ mice. Ear swelling (Figure 4a), clinical score (Figure 4b) and inflammatory cell infiltration (Figure 4c) were all reduced. The ELISA results confirmed that MC903-induced leukocyte activation related protein MPO and MMP-9, eosinophil activation-related protein eotaxin-2 were all decreased in ST2^−/−^ mice compared with BL6 mice (Figures 4d and f), as well as the Th2 cytokines IL-4, IL-13 and IL-5 (Figures 4g and i).

**Figure 5:**
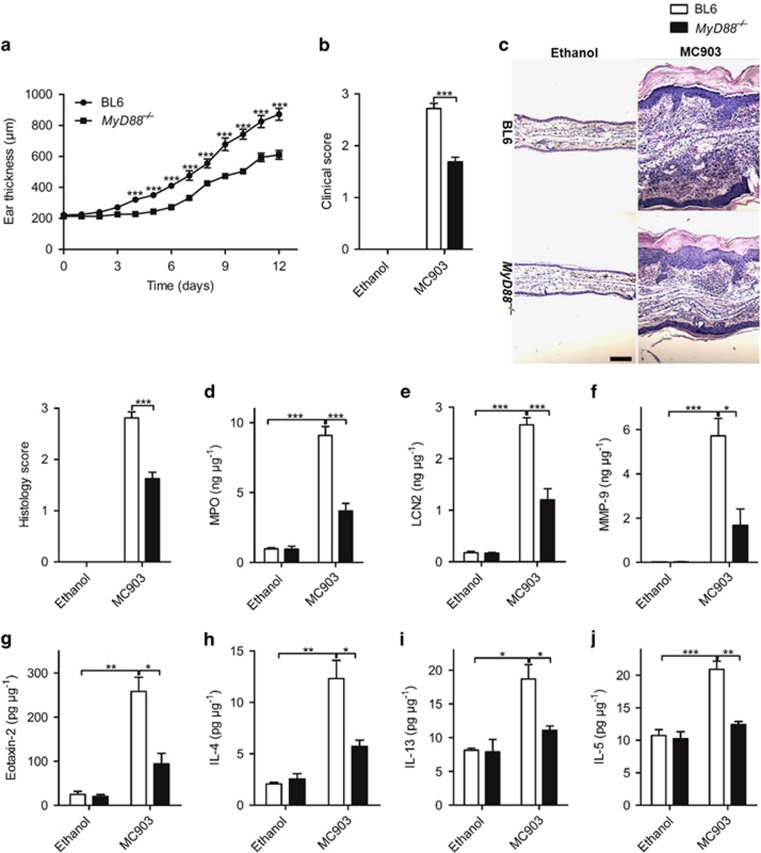
IL-33 mediates MC903-induced AD via MyD88 pathway. (**a**) The ear thickness of BL6 and MyD88^−/−^ mice after daily MC903 treatment. (**b** and **c**) Clinical score (**b**) and H&E staining of the ears (**c**) of BL6 and MyD88^−/−^ mice treated with ethanol or MC903 at day 12. Scale bar represents 50 *μ*m. (**d**-**j**) The protein expression of MPO (**d**), LCN-2 (**e**), MMP-9 (**f**), eotaxin-2 (**g**), IL-4 (**h**), IL-13 (**i**) and IL-5 (**j**) in the ear of BL6 and MyD88^−/−^ mice treated with ethanol or MC903 at day 12 *P<0.05,**P<0.01,***P<0.001. *P*-values were analyzed by two-way ANOVA in **a** and one-way ANOVA in **b**-**j**. All data are representative of two to three independent experiments with *n*=4–7 per group and are means±S.E.M.

Second, we evaluated whether the common IL-1R family adaptor protein MyD88 would be involved in the development of AD phenotype. Compared with BL6 mice, ear thickness was reduced in MyD88^−/−^ mice 12 days after daily topical MC903 administration (Figure 5a). Clinical signs of skin inflammation and inflammatory cell infiltration were drastically reduced in the absence of MyD88 (Figures 5b and c). Neutrophil recruitment and activation measured by MPO, LCN-2 and MMP-9 as well as eosinophil activation related protein eotaxin-2 expression were all significantly decreased in MyD88^−/−^ mice as compared with BL6 mice (Figures 5d and g). Consistent with reduced inflammation, the expression of Th2 cytokines IL-4, IL-13 and IL-5 was diminished (Figures 5h and j). Therefore, the data demonstrate that IL-33 mediates MC903-induced AD via ST2-MyD88 pathway.

### MyD88 signaling by DCs is critical for AD development

**Figure 6:**
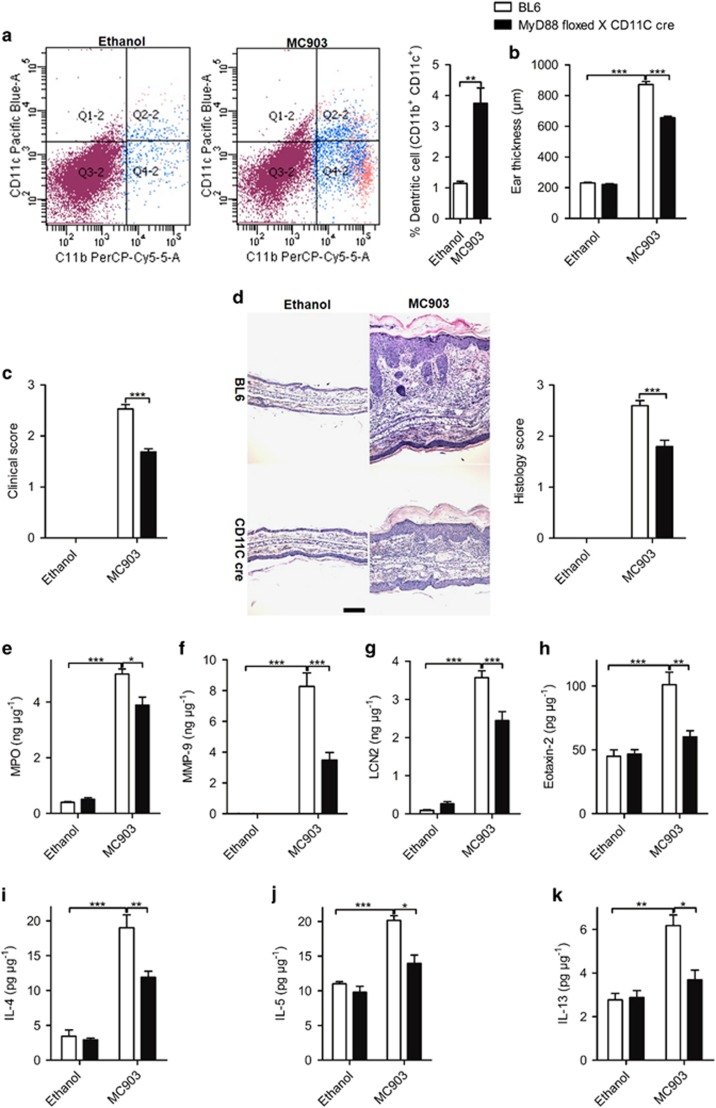
IL-33-MyD88 signaling in DCs is required for AD development. (**a**) DC infiltration in skin lesions. (**b**–**d**) The ear thickness (**b**), clinical score (**c**) and H&E staining of the ear (**d**) of BL6 and MyD88-floxed × CD11c-cre mice after 12 days of MC903 treatment. Scale bar represents 50 *μ*m. (**e**-**i**). The protein expression of MPO (**e**), MMP-9 (**f**), LCN-2 (**g**), eotaxin-2 (**h**), IL-4 (**i**), IL-5 (**j**) and IL-13 (**k**) in the ear of BL6 and MyD88-floxed × CD11c-cre mice treated with ethanol or MC903 at day 12. *P<0.05,**P<0.01,***P<0.001.*P*-values were analyzed by two-tailed *t*-tests in **a** and one-way ANOVA in **b**-**k**. All data are representative of two to three independent experiments with *n*=7-8 per group and are means±S.E.M.

IL-33 released by keratinocytes, endothelial cells and other immune cells activates ST2, followed by the activation of MyD88 and expression of factors implicated in several inflammatory pathways.^3, 24, 25, 26^ To define which cell type is involved in AD development, we used cell-specific MyD88-deficient mice using the cre-lox system^27^ and found that MC903-induced AD syndrome was drastically reduced in DC-specific MyD88 (CD11c-cre × MyD88-floxed) deficient mice. Flow cytometry revealed increased DC cells infiltration in MC903-induced skin lesions in BL6 mice (Figure 6a). Ear swelling, clinical score and inflammatory cell infiltration were significantly decreased in the absence of MyD88 in DCs (Figures 6-d). The reduction of skin inflammation was associated with decreased MPO, MMP-9, LCN-2 and eotaxin-2, (Figures 6e and h), as well as Th2 cytokines expression (Figures 6i and k). Therefore, MyD88 signaling in DCs is critical for MC903-induced AD development.

## Discussion

The IL-1 family cytokine IL-33 (IL-F11) is known to promote Th2 responses and may have an important role in the initiation of AD as the epidermal administration of IL-33 caused an AD-like inflammation.^28^ However, the function of other IL-1 family cytokines, such as IL-1*α*, IL-1*β*, IL-36*α*/*β*/*γ* and IL-36Ra, in the pathogenesis of MC903 AD was not explored before. Here we investigated the pathway of MC903-induced AD in BL6, IL-33^−/−^, IL-33 receptor ST2^−/−^, IL-36 R^−/−^, IL-36Ra^−/−^, IL-1*αβ*^−/−^, IL-1R1^−/−^ and MyD88^−/−^ mice. We report a critical role of IL-33/ST2 signaling as experimental AD in IL-33- and ST2^−/−^ mice was drastically reduced with only minor reduction in IL-1*αβ*^−/−^ mice, but no difference in IL-36 R^−/−^ and IL-36Ra^−/−^ mice. As most IL-1 family members signal through MyD88, we used MyD88^−/−^ and DC-specific MyD88^−/−^ (CD11c-cre × MyD88-floxed) deficient mice. We report that MC903-induced inflammatory response is reduced in MyD88^−/−^ mice and largely DC dependent as the AD is reduced in DC-specific MyD88-deficient mice. Therefore, we identified a critical role of IL-33 signaling via ST2 and uncovered the pivotal function of MyD88 in DCs in MC903-induced AD-like skin inflammation.

The IL-1 and IL-1 R families have grown impressively in size, complexity and division of labor. The discovery of innate lymphoid cells (ILCs) and the dissection of pathways of T-cell differentiation have revealed essential and distinctive functions for IL-1, IL-36, IL-33.^13^ IL-1*α* and IL-1*β* are encoded by distinct genes, signaling through the same receptor complex (IL-1R1 and IL-1RAcP), and have similar biological properties. IL-1 promotes T-cell responses with a key role for the differentiation of Th17 cells mediating autoimmune and chronic inflammatory diseases, such as psoriasis.^29^ Despite no much significant difference in inflammation, the expression of MMP-9, eotaxin-2, IL-4 and IL-13 was reduced in IL-1*αβ*^−/−^ and IL-1R1^−/−^ mice compared with BL6 after 12 days of MC903 application, which indicated IL-1R1 has minor role in MC903-induced AD. These results are consistent with the report that IL-1 may have a promoting role in the development of the atopic changes.^18^

IL-36 family members IL-36*α* (IL-1F6), IL-36*β* (IL-1F8) and IL-36*γ* (IL-1F9) bind to IL-1Rrp2 and use IL-1RAcP as a co-receptor. IL-36Ra (IL-1F5), which shares >50% homology with IL-1Ra, is a receptor antagonist.^13^ IL-36 is produced by innate immune cells and lymphocytes inducing the production of proinflammatory cytokines, chemokines and costimulatory molecules, thus promoting Th1 and Th17 cell polarization.^30, 31^ There is evidence that IL-36 is involved in pathological conditions including psoriasis^32^ and *A. fumigatus* infection,^33^ but little is known about the function of IL-36 in Th2 type skin diseases. Unlike to the data in experimental psoriasis, IL-36 is not essential for MC903-induced experimental AD.

IL-33 signals through the ST2 receptor, which associates with IL-1RAcP to induce MyD88-dependent signaling. IL-33 is a cytokine mainly involved in type 2 immunity and inflammation. Its main effects on innate and adaptive cells, including ILC2, Th2 cells and alternatively activated M2 polarized macrophages, are consistent with this general function.^13, 34^ Furthermore, IL-25 and IL-33 drive type 2 ILCs in AD.^35^ We reported before that DCs respond to IL-33 and contribute to Th2 differentiation in the lung.^36^ Our data demonstrated that the IL-1 family member IL-33 may be a key factor in MC903-induced experimental AD, and activate DCs favoring Th2 responses in the pathogenesis of this allergic skin disease.

DCs are highly specialized professional antigen-presenting cells and are usually located at surveillance interfaces of the human body such as the skin or mucosa, and are thought to have an important role in the initiation and regulation of immune responses, including in AD.^37, 38, 39^ Here we observed MC903-induced AD was reduced in DC-specific MyD88-deficient mice compared with BL6 mice, suggesting that MyD88 signaling in DCs is critical for AD development. Interaction between antigen-loaded DCs and antigen-specific T cells leads to T-cell proliferation and differentiation and generation of Th1, Th2, Th17 or T reg cells.^2^ In AD, there is an increased expression of Th2 cytokines, serum IgE levels and eosinophilia.^40^ Furthermore, allergen-specific T cells are increased producing IL-4, IL-5 and IL-13, but little IFN-*γ* in the peripheral blood of patients with AD.^40, 41, 42^ We found MC903-induced experimental AD was only slightly reduced in mice deficient of MyD88 signaling in T cells (MyD88-floxed × CD4-cre mice) as compared with BL6 mice (data not shown). Our data suggest that IL-33 acts through MyD88 signaling via DCs with a minor contribution by T cells during AD development. For the mechanism of IL-33 in influencing the ability of DCs to polarize T cells, we have shown before that IL-33 activated myeloid DCs to produce IL-6, IL-1*β*, TNF, CCL17 and to express high levels of CD40, CD80, OX40L and CCR7. Moreover, IL-33-activated DCs prime naive lymphocytes to produce the Th2 cytokines.^36^

ILCs are a family of developmentally related cells that are involved in immunity and in tissue development and remodeling.^43^ Three distinct members of this family have been identified on the basis of their differential developmental requirements and expression of effector cytokines.^44^ Among these, type 2 ILC2 produce the Th2-associated cytokines to induce lung inflammation in certain models of allergic asthma.^43^ Moreover, ILC2 are enriched in the lesional skin of patients with AD^44^ and skin-specific expression of IL-33 developed a spontaneous AD-like skin inflammation, which is associated with ILC2 infiltration.^45^ In this study, we found both TSLP and IL-33 were significantly upregulated in MC903-induced AD-like skin inflammatory responses. The increased inflammatory responses, including Th2 cytokine expression were significantly suppressed in IL-33^−/−^ or ST2^−/−^ mice, which means that IL-33 and its receptor ST2 are essential for MC903-induced Th2 cytokine expression. However, whether IL-33-mediated Th2 response is dependent on ILC2 needs further investigation.

In contrast to the present results and the previous findings that IL-33 was crucial for the development of AD-like inflammation in mice,^35, 45^ Kim *et al.*^44^ revealed that TSLP elicits IL-33-independent ILC2 responses to promote skin inflammation in AD model. The reasons for the discrepancy between our results and the findings from Kim *et al.* may be due to a higher dose of MC903 and earlier time point for the microscopic analysis. Importantly, TSLP expression was significantly increased at early stage (detectable even earlier than day 3 and reaching a maximum at day 6 and declined rapidly) in response to MC903, whereas IL-33 and Th2 cytokines, as well as neutrophil recruitment and activation-related mediators, such as MPO, LCN-2 and MMP-9 expression, were detectable at days 3 or 6 and reaching a maximum at day 12. Therefore, we speculate that TSLP may have a pivotal function for MC903-induced AD-like syndrome at the early stage, whereas IL-33 is essential for sustained inflammation with amplification at a later stage. Consistent with the previous report,^35^ we found that ear thickness was only slightly reduced in IL-33^−/−^ mice when compared with WT controls after 4 days of application, but continuous MC903 application augmented the ear thickness to a greater extent in WT mice than in IL-33^−/−^ mice.

Keratinocyte-derived TSLP is essential for vitamin D3- and analog-induced AD-like inflammatory responses.^20, 23^ Our results showed that the vitamin D3 analog MC903-induced gene and protein expression levels of TSLP in a time-dependent manner (Figures 1m and n). By inducing AD model in BL6 and TSLPR^−/−^ mice, we found MC903-induced AD was markedly decreased in TSLPR^−/−^ mice^20^ as compared with BL6 mice, including the inflammatory cell infiltration and Th2 cytokine expression, suggesting that TSLP is essential for MC903-induced AD, and IL-33 has a complementary function. For the mechanisms of TSLP expression, it has been demonstrated that the RXR vitamin D receptor and RXR retinoic acid receptor heterodimers and their ligand autonomously control the expression of TSLP in epidermal keratinocytes.^20^ We found time-dependent expression of IL-33 in both gene and protein after MC903 treatment. The expression of IL-33 is primarily localized to non-hematopoietic cells, particularly keratinocytes,^46^ and this upregulated IL-33 is essential for mucosal and systemic innate, rather than acquired immune responses.^47^ Consistent with the reports before, the immunofluorescence analysis showed the basal level expression of IL-33 was mainly in epidermal cells in the ethanol group, however, after 12 days of MC903 treatment, IL-33 expression was upregulated in epidermal cells and to a lesser extent in infiltrating dermal myeloid cells.

In conclusion, we found that IL-33 is largely produced by keratinocytes upon repeated cutaneous MC903 administration and AD is dependent on IL-33/ST2 signaling. Further, we uncovered a critical role of MyD88 in DCs for IL-33 signaling via ST2 in AD development. The data suggest that IL-33/ST2 may represent a therapeutic target for this chronic inflammatory disease of the skin.

## Materials and Methods

### Mice

C57BL/6 mice and age-matched IL-33^−/−^^47^, ST2^−/−^^48^, IL-36R^−/−^/IL-36Ra^−/−^^31^, *IL-1αβ*^−/−^^49^/IL-1R1^−/−^^50^, MyD88^−/−^^51^, MyD88-floxed × CD11c-cre and MyD88-floxed × CD4-cre^27^ and IL-33 citrine reporter^52^ mice (7–9 weeks old) were housed in our specific pathogen-free animal facility at CNRS (UPS 44, Transgenose Institute, Orleans, France). Mice were maintained in a temperature-controlled facility with a strict 12-h light/dark cycles and were given free access to food and water.

### Ethics statement

All animal experimental protocols complied with the French ethical and animal experiments regulations (see Charte Nationale, Code Rural R 214-122, 214-124 and European Union Directive 86/609/EEC) and were approved by the 'Ethics Committee for Animal Experimentation of CNRS Campus Orleans' (CCO), registered (N°3) by the French National Committee of Ethical Reflexion for Animal Experimentation (CLE CCO 2012-042).

### MC903-induced AD model

To induce AD lesion, 1 nmol of MC903 in 10 *μ*l ethanol or ethanol (vehicle) was painted on both ears daily for 12 days.^20, 35^ Twenty-four  hours after last challenge, mice were killed, the ear thickness was measured using a digital caliper (Decimal Caliper, Asa Dental spa, Lucca, Italy), images of the ear were taken daily and clinical score was evaluated as none (0), mild (1), moderate (2) or severe (3) according to the ear redness and scaling.

### Histology

Paraffin-embedded ear sections were used by haematoxylin–eosin staining for the histopathological diagnosis, and the clinical histology score was evaluated as none (0), mild (1), moderate (2) or severe (3) according to the dermal cell infiltration, epidermal hyperplasia and scurf production, the analysis was performed using conventional optical microscopy (Leica, Wetzlar, Germany).

### Real-time quantitative RT-PCR

Total RNA was prepared using Trizol Reagent (Invitrogen, Carlsbad, CA, USA) following the manufacturer's instructions. RNA was quantified by Thermo NANODROP 2000 spectrophotometer (Thermo Fisher Scientific, Waltham, MA), total RNA (1 *μ*g) was reverse transcribed using GoScript Reverse Transcription System (Promega, Madison, WI, USA, cat: A5001) according to the manufacturer's instructions. Q-PCR were performed on Mx3005P (Stratagene, La Jolla, CA, USA) using GoTaq q-PCR Master Mix (Promega, cat: A6001/2). The following gene expression assays were purchased from Qiagen (Hilden, Germany): *GAPDH* (NM_001289726.1; cat: QT01658692), *IL-33* (NM_133775.2; cat: QT00135170)*, IL-1α* (NM_010554.4; cat: QT00113505)*, IL-1β* (NM_008361.4; cat: QT01048355), *IL-36α* (NM_019450.3; cat: QT00136745), *IL-36γ* (NM_153511.3; cat: QT00146734), *IL-36Ra* (cat: QT00252931). The primer for *IL-36β*_(NM_027163.4) is purchased from Sigma-Aldrich (St. Louis, MO, USA) with the sequence: 5′-ACAAAAAGCCTTTCTGTTCT-3′ (forward) and 5′-CCATGTTGGATTTACTTCTC-3′ (reverse). Quantification of gene expression was determined by the comparative 2^ΔΔ*CT*^ method. The relative expression levels were determined by normalizing expression to glyceraldehyde 3-phosphate dehydrogenase (GAPDH). All the assays were performed in triplicate and repeated at least two times.

### FACS analysis

In order to characterize the cells recruited in MC903-induced AD model, ears and lymph nodes of four BL6 mice treated with ethanol or MC903 were collected and digested in 3 ml of reagent (RPMI) containing 2 mg/ml collagenase type IV and 1 mg/ml DNase (Sigma) for 60 min at 37 °C. The suspension was then passed through a cell strainer (100 *μ*m) and washed with FACS buffer. Finally, a preparation about 10^6^ cells was prepared and stained with antibodies against mouse CD8 APC (53-6.7), CD4 V500 (RM4-5), CD11b Percp (M1/70), CD11c V450 (HL3) and Ly6G FITC (1A8) (all BD Biosciences, San Jose, CA, USA). Cells were washed with PBS EDTA, 7-AAD (BD Biosciences) was added 5 min before analysis for dead cell exclusion and analyzed using an FACS Canto II machine (BD Biosciences). FACS data were analyzed using the FlowJo Software (TreeStar, Ashland, OR, USA).

### Protein detection in the ear

Ear samples were homogenized in 1 ml PBS using Ultra Turrax (IKA-Werke, Staufen, Germany), the supernatant was harvested and assayed for cytokine content using commercially available enzyme-linked immunosorbent assay reagents for MPO, MMP-9, LCN-2, eotaxin-2, IL-33, IL-10, IL-1*γ*, IL-4, IL-13 and IL-5. (Duoset R&D Systems, Abingdon, UK).

### Cell culture and stimulation

Neonatal human epidermal keratinocytes (Cascade Biologics, Portland, OR, USA) were cultured in serum-free EpiLife medium (Cascade Biologics) containing 0.6 mM Ca^2+^, 1 × Epilife defined growth supplement, 50 U/ml penicillin and 50 *μ*g/ml streptomycin under standard culture conditions. Murine primary keratinocytes were isolated from newborn skin as using dispase II and cultured in 154CF medium supplemented with HKGS, 0.2 mM CaCl_2_ (Invitrogen) and Pen Strep (100 units/ml penicillin and 100ug/ml sreptomycin) (Invitrogen). Primary dermal fibroblasts were isolated from newborn mice using dispase _ Σ. Following skin digestion, isolated cells were centrifuged after filtering and plated with DMEM (GIBCO, Carlsbad, CA, USA) containing 10% FBS and 1% penicillin/streptomycin. Primary peritoneal neutrophils were isolated from six BL6 mice by i.p. injection of 3 ml thioglycolate medium (Sigma). Cells were harvested 4 h later by peritoneal lavage with cold PBS, followed by washing with RPMI1640 medium (GIBCO). Bone marrow-derived macrophages and DCs from four BL6 mice were cultured as previously reported.^27, 36^ RAW264.7 cells and human fibroblast cell lines (MEF) (Chinese Academy of Sciences, Shanghai, China) were cultured in DMEM (Invitrogen) medium containing 10% FBS (GIBCO), 50 U/ml penicillin and 50 ug/ml streptomycin (GIBCO) under standard culture conditions. For all cell stimulation experiments, 2 × 10 ^5^ cells were seeded in each well of 24-well or 8 × 10^5^ cells were seeded in each well of 6-well plates. When cells were grown to 80% confluence, the indicated doses of MC903 were used to stimulate cells for different times. After treatment, cells were harvested in Trizol for RNA extraction and cell lysates were harvested in RIPA buffer for western analysis.

### Immunofluorescent staining

Skin from MC903-treated mice was frozen, cut at 7 *μ*m on a cryotome, mounted on glass slides, fixed in 4% PFA for 10 min and subsequent pretreated with antigen retrieval solution for 10 min at 95 °C. The sections were then stained with goat anti-mouse IL-33 antibody (cat: AF3626, R&D Systems), incubated at 4 °C overnight. The sections were incubated with FITC-conjugated donkey anti-goat antibody (R&D Systems) for 1 h at RT and then mounted in ProLong Gold antifade reagent with DAPI (Invitrogen) and visualized them by the microscope (Leica). For the IL-33 protein detection in the skin of IL-33 citrine reporter mice was done according to our previous report.^52^

### Statistical analysis

All data are present as mean±S.E.M. We used two-tailed *t*-tests to determine significances between two groups. We did analyses of multiple groups by one-way or two-way ANOVA with Bonferroni post test of GraphPad prism version 5 (Graphpad Software, La Jolla, CA, USA). For all statistical tests, we considered *P*-value <0.05 to be statistically significant.

## References

[bib1] Leung DY, Bieber T. Atopic dermatitis. Lancet 2003; 361: 151–160. 12531593 10.1016/S0140-6736(03)12193-9

[bib2] Jin H, He R, Oyoshi M, Geha RS. Animal models of atopic dermatitis. J Invest Dermatol 2009; 129: 31–40. 19078986 10.1038/jid.2008.106 PMC2886143

[bib3] Cevikbas F, Steinhoff M. IL-33: a novel danger signal system in atopic dermatitis. J Invest Dermatol 2012; 132: 1326–1329. 22499037 10.1038/jid.2012.66 PMC3694595

[bib4] Leung DY, Boguniewicz M, Howell MD, Nomura I, Hamid QA. New insights into atopic dermatitis. J Clin Invest 2004; 113: 651–657. 14991059 10.1172/JCI21060 PMC351324

[bib5] Novak N, Bieber T, Leung DY. Immune mechanisms leading to atopic dermatitis. J Allergy Clin Immunol 2003; 112(6 Suppl): S128–S139. 14657843 10.1016/j.jaci.2003.09.032

[bib6] Wollenberg A, Feichtner K. Atopic dermatitis and skin allergies - update and outlook. Allergy 2013; 68: 1509–1519. 24410780 10.1111/all.12324

[bib7] Boguniewicz M, Leung DY. Atopic dermatitis: a disease of altered skin barrier and immune dysregulation. Immunol Rev 2011; 242: 233–246. 21682749 10.1111/j.1600-065X.2011.01027.x PMC3122139

[bib8] Brown SJ, McLean WH. Eczema genetics: current state of knowledge and future goals. J Invest Dermatol 2009; 129: 543–552. 19209157 10.1038/jid.2008.413

[bib9] Thomsen SF. Atopic dermatitis: natural history, diagnosis, and treatment. ISRN Allergy 2014; 2014: 354250. 25006501 10.1155/2014/354250 PMC4004110

[bib10] Lund S, Walford HH, Doherty TA. Type 2 innate lymphoid cells in allergic disease. Curr Immunol Rev 2013; 9: 214–221. 24876829 10.2174/1573395510666140304235916 PMC4033554

[bib11] Liu FT, Goodarzi H, Chen HY. IgE, mast cells, and eosinophils in atopic dermatitis. Clin Rev Allergy Immunol 2011; 41: 298–310. 21249468 10.1007/s12016-011-8252-4

[bib12] Levin J, Fallon Friedlander S, Del Rosso JQ. Atopic dermatitis and the stratum corneum: part 3: the immune system in atopic dermatitis. J Clin Aesthet Dermatol 2013; 6: 37–44. PMC3997207 24765223

[bib13] Garlanda C, Dinarello CA, Mantovani A. The interleukin-1 family: back to the future. Immunity 2013; 39: 1003–1018. 24332029 10.1016/j.immuni.2013.11.010 PMC3933951

[bib14] Dinarello CA. Anti-inflammatory agents: present and future. Cell 2010; 140: 935–950. 20303881 10.1016/j.cell.2010.02.043 PMC3752337

[bib15] Dinarello CA. Interleukin-1 in the pathogenesis and treatment of inflammatory diseases. Blood 2011; 117: 3720–3732. 21304099 10.1182/blood-2010-07-273417 PMC3083294

[bib16] Dinarello C, Arend W, Sims J, Smith D, Blumberg H, O'Neill L et al. IL-1 family nomenclature. Nat Immunol 2010; 11: 973. 20959797 10.1038/ni1110-973 PMC4174560

[bib17] Savinko T, Matikainen S, Saarialho-Kere U, Lehto M, Wang G, Lehtimaki S et al. IL-33 and ST2 in atopic dermatitis: expression profiles and modulation by triggering factors. J Invest Dermatol 2012; 132: 1392–1400. 22277940 10.1038/jid.2011.446

[bib18] Konishi H, Tsutsui H, Murakami T, Yumikura-Futatsugi S, Yamanaka K, Tanaka M et al. IL-18 contributes to the spontaneous development of atopic dermatitis-like inflammatory skin lesion independently of IgE/stat6 under specific pathogen-free conditions. Proc Natl Acad Sci USA 2002; 99: 11340–11345. 12151598 10.1073/pnas.152337799 PMC123258

[bib19] Elentner A, Finke D, Schmuth M, Chappaz S, Ebner S, Malissen B et al. Langerhans cells are critical in the development of atopic dermatitis-like inflammation and symptoms in mice. J Cell Mol Med 2009; 13: 2658–2672. 19538461 10.1111/j.1582-4934.2009.00797.x PMC8183941

[bib20] Li M, Hener P, Zhang Z, Kato S, Metzger D, Chambon P. Topical vitamin D3 and low-calcemic analogs induce thymic stromal lymphopoietin in mouse keratinocytes and trigger an atopic dermatitis. Proc Natl Acad Sci USA 2006; 103: 11736–11741. 16880407 10.1073/pnas.0604575103 PMC1544239

[bib21] Choi J, Kim JR, Kim H, Kim YA, Lee HJ, Kim J et al. The atopic dermatitis-like symptoms induced by MC903 were alleviated in JNK1 knockout mice. Toxicol Sci 2013; 136: 443–449. 24046278 10.1093/toxsci/kft215

[bib22] Kapp A. The role of eosinophils in the pathogenesis of atopic dermatitis—eosinophil granule proteins as markers of disease activity. Allergy 1993; 48: 1–5. 10.1111/j.1398-9995.1993.tb02167.x 8457021

[bib23] Li M, Hener P, Zhang Z, Ganti KP, Metzger D, Chambon P. Induction of thymic stromal lymphopoietin expression in keratinocytes is necessary for generating an atopic dermatitis upon application of the active vitamin D3 analogue MC903 on mouse skin. J Invest Dermatol 2009; 129: 498–502. 18650845 10.1038/jid.2008.232

[bib24] Pushparaj PN, Tay HK, H'Ng SC, Pitman N, Xu D, McKenzie A et al. The cytokine interleukin-33 mediates anaphylactic shock. Proc Natl Acad Sci USA 2009; 106: 9773–9778. 19506243 10.1073/pnas.0901206106 PMC2700978

[bib25] Li C, Li H, Jiang Z, Zhang T, Wang Y, Li Z et al. Interleukin-33 increases antibacterial defense by activation of inducible nitric oxide synthase in skin. PLoS Pathog 2014; 10: e1003918. 24586149 10.1371/journal.ppat.1003918 PMC3930573

[bib26] Liew FY, Pitman NI, McInnes IB. Disease-associated functions of IL-33: the new kid in the IL-1 family. Nat Rev Immunol 2010; 10: 103–110. 20081870 10.1038/nri2692

[bib27] Agoro R, Piotet-Morin J, Palomo J, Michaudel C, Vigne S, Maillet I et al. IL-1R1-MyD88 axis elicits papain-induced lung inflammation. Eur J Immunol 2016; 46: 2531–2541. 27569535 10.1002/eji.201646366

[bib28] Hueber AJ, Alves-Filho JC, Asquith DL, Michels C, Millar NL, Reilly JH et al. IL-33 induces skin inflammation with mast cell and neutrophil activation. Eur J Immunol 2011; 41: 2229–2237. 21674479 10.1002/eji.201041360

[bib29] Sims JE, Smith DE. The IL-1 family: regulators of immunity. Nat Rev Immunol 2010; 10: 89–102. 20081871 10.1038/nri2691

[bib30] Vigne S, Palmer G, Lamacchia C, Martin P, Talabot-Ayer D, Rodriguez E et al. IL-36R ligands are potent regulators of dendritic and T cells. Blood 2011; 118: 5813–5823. 21860022 10.1182/blood-2011-05-356873

[bib31] Vigne S, Palmer G, Martin P, Lamacchia C, Strebel D, Rodriguez E et al. IL-36 signaling amplifies Th1 responses by enhancing proliferation and Th1 polarization of naive CD4+ T cells. Blood 2012; 120: 3478–3487. 22968459 10.1182/blood-2012-06-439026

[bib32] Tortola L, Rosenwald E, Abel B, Blumberg H, Schafer M, Coyle AJ et al. Psoriasiform dermatitis is driven by IL-36-mediated DC-keratinocyte crosstalk. J Clin Invest 2012; 122: 3965–3976. 23064362 10.1172/JCI63451 PMC3484446

[bib33] Blumberg H, Dinh H, Trueblood ES, Pretorius J, Kugler D, Weng N et al. Opposing activities of two novel members of the IL-1 ligand family regulate skin inflammation. J Exp Med 2007; 204: 2603–2614. 17908936 10.1084/jem.20070157 PMC2118475

[bib34] Liew FY, Girard JP, Turnquist HR. Interleukin-33 in health and disease. Nat Rev Immunol 2016; 16: 676–689. 27640624 10.1038/nri.2016.95

[bib35] Salimi M, Barlow JL, Saunders SP, Xue L, Gutowska-Owsiak D, Wang X et al. A role for IL-25 and IL-33-driven type-2 innate lymphoid cells in atopic dermatitis. J Exp Med 2013; 210: 2939–2950. 24323357 10.1084/jem.20130351 PMC3865470

[bib36] Besnard AG, Togbe D, Guillou N, Erard F, Quesniaux V, Ryffel B. IL-33-activated dendritic cells are critical for allergic airway inflammation. Eur J Immunol 2011; 41: 1675–1686. 21469105 10.1002/eji.201041033

[bib37] Novak N, Bieber T. The role of dendritic cell subtypes in the pathophysiology of atopic dermatitis. J Am Acad Dermatol 2005; 53(2 Suppl 2): S171–S176. 16021172 10.1016/j.jaad.2005.04.060

[bib38] Wollenberg A, Wagner M, Gunther S, Towarowski A, Tuma E, Moderer M et al. Plasmacytoid dendritic cells: a new cutaneous dendritic cell subset with distinct role in inflammatory skin diseases. J Invest Dermatol 2002; 119: 1096–1102. 12445198 10.1046/j.1523-1747.2002.19515.x

[bib39] Wuthrich B, Schmid-Grendelmeier P. The atopic eczema/dermatitis syndrome. Epidemiology, natural course, and immunology of the IgE-associated ("extrinsic") and the nonallergic ("intrinsic") AEDS. J Investig Allergol Clin Immunol 2003; 13: 1–5. 12861844

[bib40] Leung DY. Atopic dermatitis: new insights and opportunities for therapeutic intervention. J Allergy Clin Immunol 2000; 105: 860–876. 10808164 10.1067/mai.2000.106484

[bib41] Kimura M, Tsuruta S, Yoshida T. Unique profile of IL-4 and IFN-gamma production by peripheral blood mononuclear cells in infants with atopic dermatitis. J Allergy Clin Immunol 1998; 102: 238–244. 9723667 10.1016/s0091-6749(98)70092-2

[bib42] Kimura M, Tsuruta S, Yoshida T. Correlation of house dust mite-specific lymphocyte proliferation with IL-5 production, eosinophilia, and the severity of symptoms in infants with atopic dermatitis. J Allergy Clin Immunol 1998; 101(1 Pt 1): 84–89. 9449505 10.1016/S0091-6749(98)70197-6

[bib43] Spits H, Artis D, Colonna M, Diefenbach A, Di Santo JP, Eberl G et al. Innate lymphoid cells—a proposal for uniform nomenclature. Nat Rev Immunol 2013; 13: 145–149. 23348417 10.1038/nri3365

[bib44] Kim BS, Siracusa MC, Saenz SA, Noti M, Monticelli LA, Sonnenberg GF et al. TSLP elicits IL-33-independent innate lymphoid cell responses to promote skin inflammation. Sci Transl Med 2013; 5: 170ra116. 10.1126/scitranslmed.3005374 PMC3637661 23363980

[bib45] Imai Y, Yasuda K, Sakaguchi Y, Haneda T, Mizutani H, Yoshimoto T et al. Skin-specific expression of IL-33 activates group 2 innate lymphoid cells and elicits atopic dermatitis-like inflammation in mice. Proc Natl Acad Sci USA 2013; 110: 13921–13926. 23918359 10.1073/pnas.1307321110 PMC3752227

[bib46] Carriere V, Roussel L, Ortega N, Lacorre DA, Americh L, Aguilar L et al. IL-33, the IL-1-like cytokine ligand for ST2 receptor, is a chromatin-associated nuclear factor *in vivo*. Proc Natl Acad Sci USA 2007; 104: 282–287. 17185418 10.1073/pnas.0606854104 PMC1765450

[bib47] Oboki K, Ohno T, Kajiwara N, Arae K, Morita H, Ishii A et al. IL-33 is a crucial amplifier of innate rather than acquired immunity. Proc Natl Acad Sci USA 2010; 107: 18581–18586. 20937871 10.1073/pnas.1003059107 PMC2972966

[bib48] Townsend MJ, Fallon PG, Matthews DJ, Jolin HE, McKenzie AN. T1/ST2-deficient mice demonstrate the importance of T1/ST2 in developing primary T helper cell type 2 responses. J Exp Med 2000; 191: 1069–1076. 10727469 10.1084/jem.191.6.1069 PMC2193113

[bib49] Labow M, Shuster D, Zetterstrom M, Nunes P, Terry R, Cullinan EB et al. Absence of IL-1 signaling and reduced inflammatory response in IL-1 type I receptor-deficient mice. J Immunol 1997; 159: 2452–2461. 9278338

[bib50] Horai R, Asano M, Sudo K, Kanuka H, Suzuki M, Nishihara M et al. Production of mice deficient in genes for interleukin (IL)-1alpha, IL-1beta, IL-1alpha/beta, and IL-1 receptor antagonist shows that IL-1beta is crucial in turpentine-induced fever development and glucocorticoid secretion. J Exp Med 1998; 187: 1463–1475. 9565638 10.1084/jem.187.9.1463 PMC2212263

[bib51] Kawai T, Adachi O, Ogawa T, Takeda K, Akira S. Unresponsiveness of MyD88-deficient mice to endotoxin. Immunity 1999; 11: 115–122. 10435584 10.1016/s1074-7613(00)80086-2

[bib52] Hardman CS, Panova V, McKenzie AN. IL-33 citrine reporter mice reveal the temporal and spatial expression of IL-33 during allergic lung inflammation. Eur J Immunol 2013; 43: 488–498. 23169007 10.1002/eji.201242863 PMC3734634

