# Supplementary material for: Experimental atopic dermatitis depends on IL-33R signaling via MyD88 in dendritic cells
Source: Cell Death Dis. 2017 Apr 6;8(4):e2735–. doi: 10.1038/cddis.2017.90 (PMC5477596; doi:10.1038/cddis.2017.90)
Supplement: Supplementary Information [file cddis201790x1.pdf]

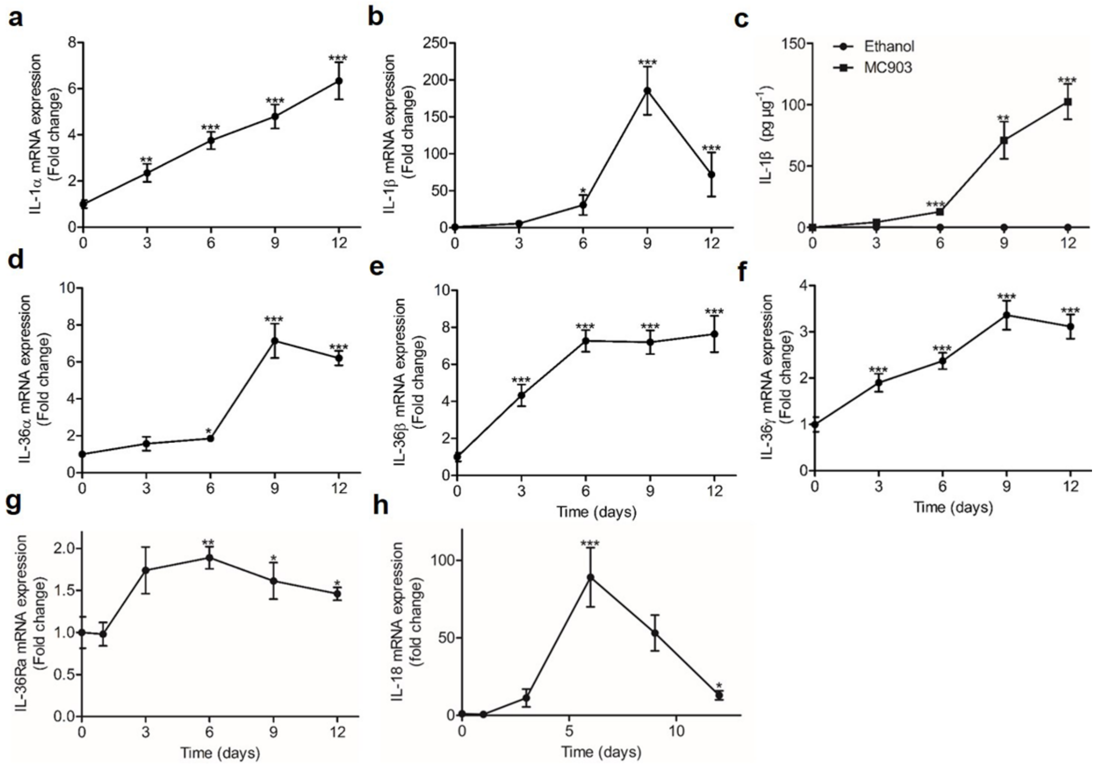

Supplementary figure 1. Expression of IL-1 and IL-36 induced by MC903. (a&b) Quantification of *IL-1α* (a) and *IL-1β* (b) mRNA expression in BL6 mouse ears for different times. (c) IL-1β protein expression in ears treated by ethanol or MC903. (d-g) Quantification of *IL-36α* (d), *IL-36β* (e), *IL-36γ* (f), *IL-36Ra* (g) and *IL-18* expression in BL6 mouse ears for different times. **P<0.05, **P<0.01, ***P<0.001*. P values were analyzed by one-way ANOVA in (a&b, d-g) or two-way ANOVA in (c). All data are representative of two to three independent experiments with n=4-5 per group and are means ± SEM.

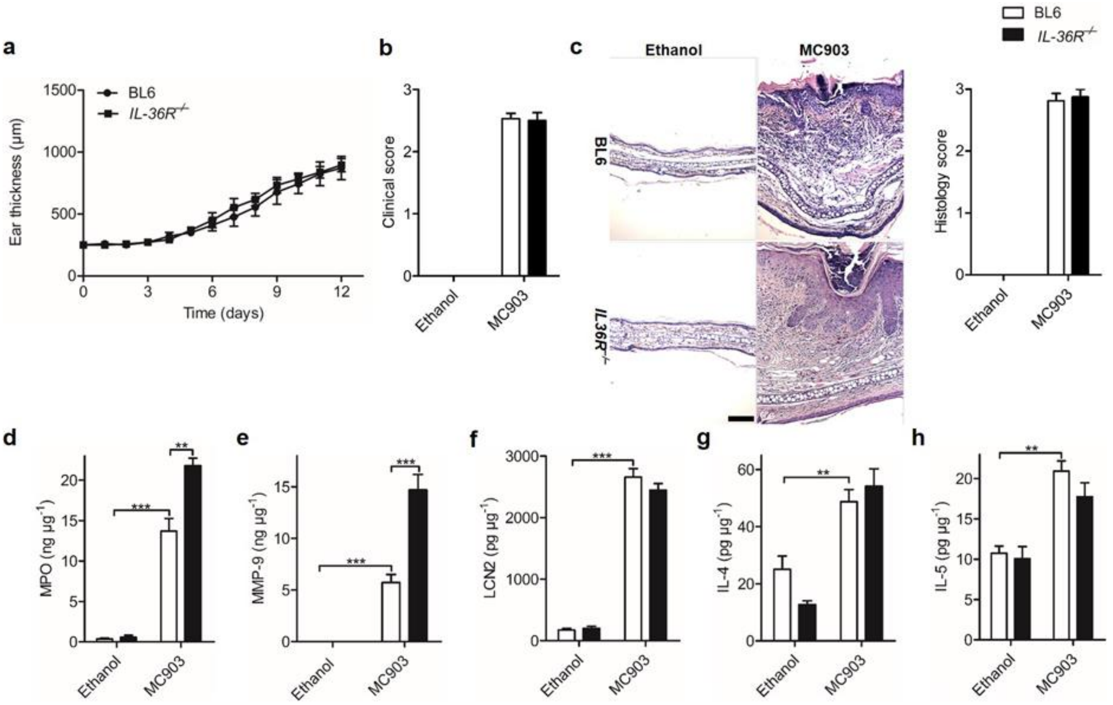

Supplementary figure 2. IL-36R signal pathway is not essential for MC903-induced AD syndrome. (a) The ear thickness of BL6 and IL-36R$^{-/-}$ mice after daily MC903 treatment. (b&c) Clinical score of the ear redness and scaling (b) and H&E staining of the ears (c) of BL6 and IL-36R$^{-/-}$ mice treated by ethanol or MC903 at day 12. Scale bar represents 50μm. (d-h) The protein expression of MPO (d), MMP-9 (e), LCN2 (f), IL-4 (g) and IL-5 (h) in the ear of BL6 and IL-36R$^{-/-}$ mice treated by ethanol or MC903 at day 12. ***P<0.01, ***P<0.001*. P values were analyzed by one-way ANOVA in (d-h). All data are representative of two to three independent experiments with n=4-7 per group and are means ± SEM.

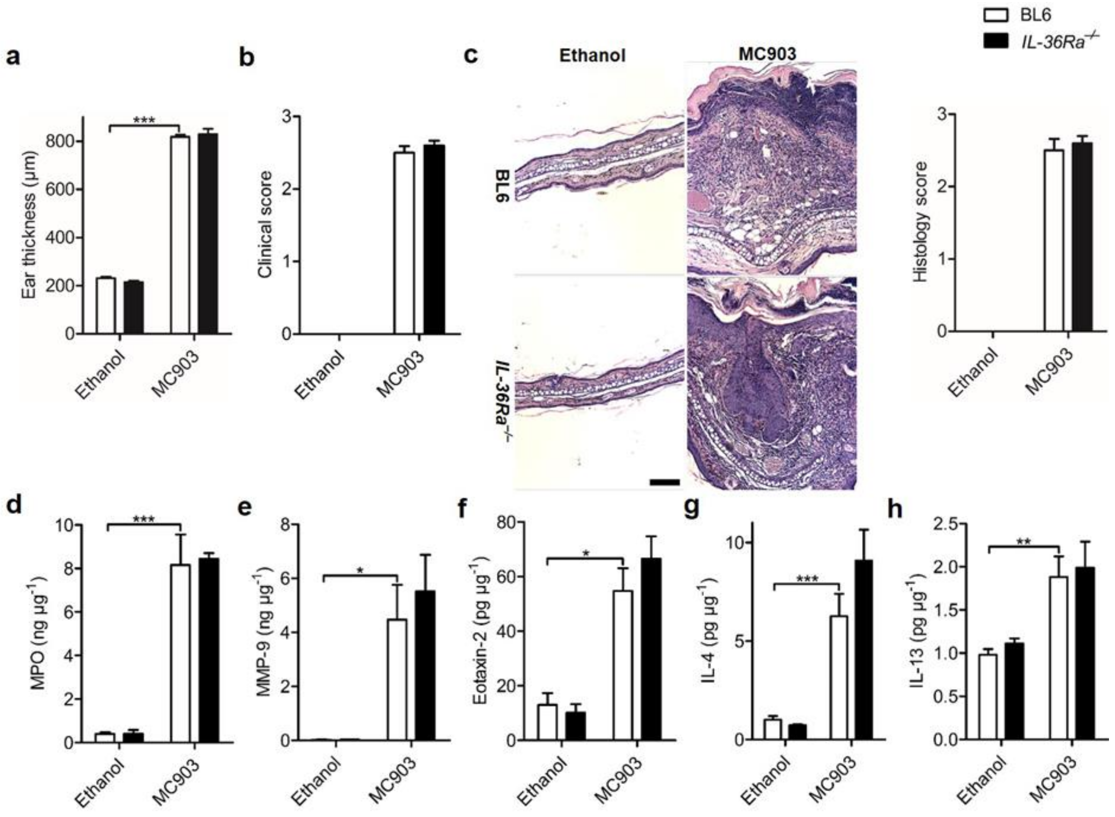

Supplementary figure 3 IL-36Ra signal pathway is not essential for MC903-induced AD syndrome. (a-c) Ear thickness (a), clinical score of ear redness and scaling (b) and H&E staining of the ears (c) of BL6 and *IL-36Ra*$^{-/-}$ mice treated by Ethanol or MC903 at day 12. Scale bar represents 50μm. (d-h) The protein expression of MPO (d), MMP-9 (e), eotaxin-2 (f), IL-4 (g) and IL-13 (h) in the ears of BL6 and IL-36Ra$^{-/-}$ mice treated by ethanol or MC903 at day 12. ***P<0.01, ***P<0.001*. P values were analyzed by one-way ANOVA in (d-h). All data are representative of two to three independent experiments with n=4-7 per group and are means ± SEM.

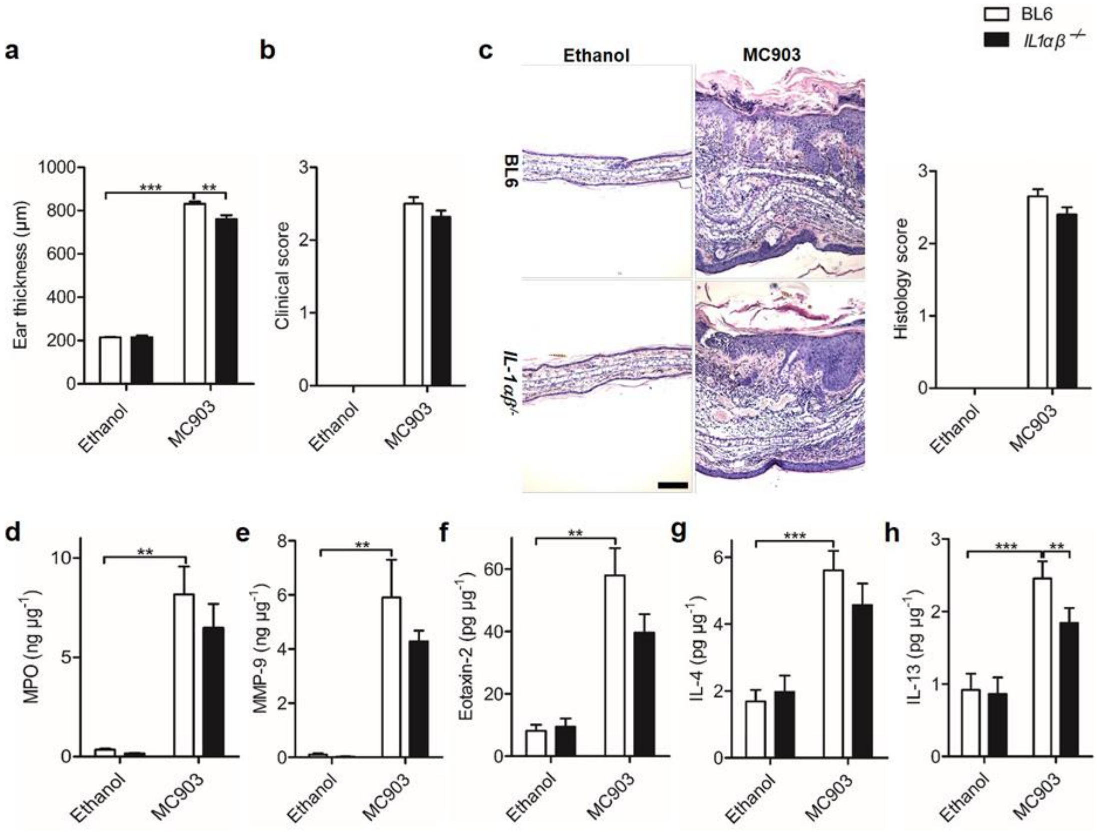

Supplementary figure 4. IL-1α/β plays a minor role in MC903-induced AD syndrome. (a-c) Ear thickness (a), clinical score of ear redness and scaling (b) and H&E staining of the ears (c) of BL6 and *IL-1αβ* $^{-/-}$ mice treated by ethanol or MC903 at day 12. Scale bar represents 50μm. (d-h) The protein expression of MPO (d), MMP-9 (e), eotaxin-2 (f), IL-4 (g) and IL-13 (h) in the ear of BL6 and *IL-1αβ* $^{-/-}$ mice treated by Ethanol or MC903 at day 12. ***P<0.01, ***P<0.001*. P values were analyzed by two-way ANOVA in (a, d-h). All data are representative of two to three independent experiments with n=7-8 per group and are means ± SEM.

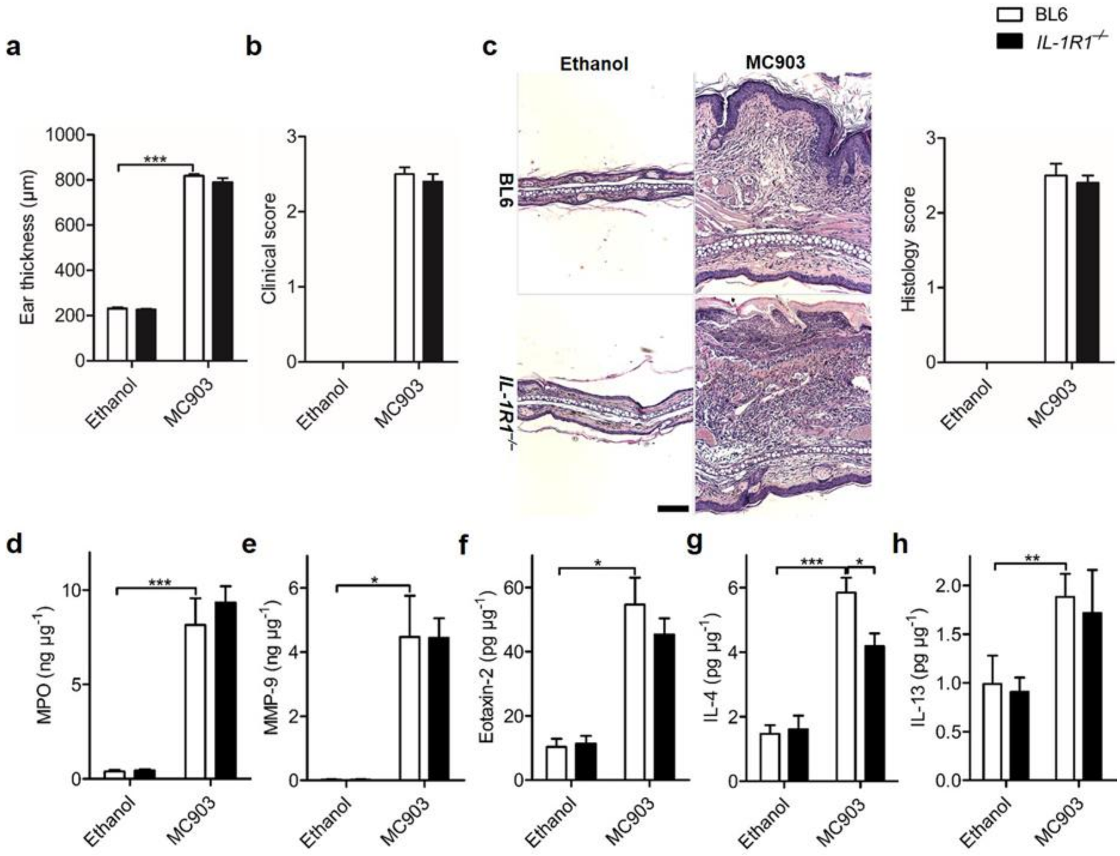

Supplementary figure 5. IL-1R signal pathway plays a minor role in MC903-induced AD syndrome. (a-c) Ear thickness (a), clinical score of ear redness and scaling (b) and H&E staining of the ears (c) of BL6 and *IL-1R1*$^{-/-}$ mice treated by Ethanol or MC903 at day 12. Scale bar represents 50μm. (d-h). The protein level of MPO (d), MMP-9 (e), eotaxin-2 (f), IL-4 (g) and IL-13 (h) in the ear of BL6 and *IL-1R1* $^{-/-}$ mice treated by Ethanol or MC903 at day 12. **P<0.05, **P<0.01, ***P<0.001*. P values were analyzed by two-way ANOVA in (a, d-h). All data are representative of two to three independent experiments with n=7-8 per group and are means ± SEM**.**
